# DISTINCT CELL SUBTYPES WITHIN NAÏVE CD4 T CELLS COMPARTMENT SHAPE ITS AGING AND SENESCENCE

**DOI:** 10.1093/geroni/igae098.2290

**Published:** 2024-12-31

**Authors:** C J Kelly, Ashley Jeon, Jacob Rodriguez, Dina Prosser, Morgan Diegel, Nikolay Burnaevskiy

**Affiliations:** Altius Institute for Biomedical Sciences, Seattle, Washington, United States; Altius Institute for Biomedical Sciences, Seattle, Washington, United States; Altius Institute for Biomedical Sciences, Seattle, Washington, United States; Altius Institute for Biomedical Sciences, Settle, Washington, United States; Altius Institute for Biomedical Sciences, Seattle, Washington, United States; Altius Institute for Biomedical Sciences, Seattle, Washington, United States

## Abstract

Aging is a multifaceted process that manifests differently in various cell types and within the same cell type. Therefore, understanding age-related changes with single-cell resolution may illuminate the mechanistic details of tissue aging and offer targets for intervention. We utilized high-throughput single-cell RNA-sequencing (scRNA-seq) to analyze senescence-associated changes within the CD4 T cell compartment. First, we sequenced total human CD4 T cells from multiple donors, revealing that naïve CD4 T cells exhibited a less senescent state compared to memory cells. We focused on naïve CD4 T cells to determine if individual cells differ in expression of aging-associated markers. By sorting cells based on senescence-associated beta-galactosidase (B-gal) activity and applying scRNA-seq, we identified transcriptional signatures associated with more youthful and senescent states. Surprisingly, we discovered cells with lower B-gal expression comprised two distinct populations: one marked by high expression of HELIOS and low expression of SESN3, and another marked by high expression of BACH2 and low expression of TC2N. In comparison, 2 youthful subpopulation and senescence subpopulations differ in markers of immunological activation We then investigated if the composition of naive CD4 compartment changes with age, finding that the abundance of youthful subpopulations was reduced in the older donors’ CD4 populations. Thus, aging in the naïve CD4 compartment is associated with a shifted distribution between more youthful and more senescent cells. Moving forward, we will attempt to manipulate naïve CD4 T cells into a more youthful state by altering expression of the identified transcriptional signatures associated with youthful and senescent states.

